# The Role of M2000 as an Anti-inflammatory Agent in Toll-Like Receptor 2/microRNA-155 Pathway

**Published:** 2017

**Authors:** Fatemeh Pourgholi, Mahsa Hajivalili, Rasoul Razavi, Shadi Esmaeili, Behzad Baradaran, Ali Akbar Movasaghpour, Sanam Sadreddini, Hamidreza Goodarzynejad, Abbas Mirshafiey, Mehdi Yousefi

**Affiliations:** 1. Hematology and Oncology Research Center, Tabriz University of Medical Sciences, Tabriz, Iran; 2. Immunology Research Center, Tabriz University of Medical Sciences, Tabriz, Iran; 3. Department of Immunology, Faculty of Medicine, Tabriz University of Medical Sciences, Tabriz, Iran; 4. Department of Hematology and Blood Banking, Tarbiat Modares University, Tehran, Iran; 5. Basic and Clinical Research Department, Tehran Heart Center, Tehran, Iran; 6. Department of Immunology, Faculty of Public Health, Tehran University of Medical Sciences, Tehran, Iran

**Keywords:** MicroRNAs, SHIP1, SOCS1

## Abstract

**Background:**

M2000 is a newly designed and safe Non-Steroidal Anti-Inflammatory Drug (NSAID). The aim of this study was to assess the effects of M2000 on expression levels of Suppressor of Cytokine Signaling-1 (SOCS-1) and Src Homology-2 domain-containing inositol-5′-phosphatase 1 (SHIP1) proteins *via* Toll-Like Receptor (TLR) 2/microRNA-155 pathway.

**Methods:**

HEK293 TLR2 cell line and Peripheral Blood Mononuclear Cells (PBMCs) were treated by different concentrations of M2000 in MTT assay. RNA was extracted by miRNeasy Mini kit. Then, cDNA was synthesized and the expression levels of SOCS1, SHIP1 and miRNA155 were evaluated by Quantitative Real time PCR.

**Results:**

Our results showed that M2000 significantly increased the expression levels of SOCS1 and SHIP-1 in Lipopolysachride (LPS)-treated and non-treated cells. Moreover, M2000 decreased expression level of miR-155 in LPS treated PBMCs.

**Conclusion:**

M2000 can be used as NSAID in LPS induced inflammation and decrease inflammatory cytokines production by targeting SOCS1, SHIP1 and miR-155 in auto-immune and inflammatory diseases.

## Introduction

Remarkable cardiovascular, gastrointestinal (GI) and renal toxicities of Non-Steroidal Anti-Inflammatory Drugs (NSAIDs) restrict their usage 1–3. In recent decades, researchers have attempted to discover safer and more effective types of NSAIDs. M2000, a novel NSAID with an uronic acid molecular structure and a low molecular weight, has no known toxicity effects on GI tract and kidney function in various experimental models 4. It was previously shown that M2000 is a powerful Matrix Metalloproteinase (MMP) inhibitor providing a new approach to the treatment of inflammatory diseases 5. Given the molecular structure of M2000, its most probable cell surface receptor belongs to mannose receptor (MR) family, and likely secondary receptors for this molecule could be Toll-Like Receptor (TLR)2, TLR4 and CD11b/CD18 (Mac-1 or CR3)^6^.

TLRs regulate a pro-/anti-inflammatory balance; among them, TLR2 has a wide range of ligands and can activate different signaling pathways 7. The Suppressors of Cytokine Signaling (SOCS) family of proteins are key components of the negative feedback loop that regulates the severity, duration and rate of cytokine signaling 8. SOCS1 is a negative regulator of inflammation, which is induced by Lipopolysaccharide (LPS) and inhibits the production of proinflammatory cytokines such as, INF-γ, IL-6 and TNF-α^9^. Another target in TLR’s signaling pathways is Src Homology-2 domain-containing inositol-5′-phosphatase 1 (SHIP1) which negatively regulates the activation of immune cells mainly *via* the phosphoinositide 3-kinase (PI-3K) pathway. Thus, down regulation of these mediators allows inflammation to proceed 10.

MicroRNA (miRNAs)-155, encoded by a noncoding gene known as BIC, B-cell integration cluster 11, is expressed in a variety of immune cells such as, T cells, macrophages, dendritic cells and B cells 12. Recently, miRNA-155 has been considered as an important regulator of innate immunity and TLR signaling pathway by targeting SOCS1 and SHIP1 affecting proinflammatory responses 13. The aim of this study was to evaluate the effects of M2000 on expression levels of SOCS1 and SHIP1 *via* TLR2 signaling pathways.

## Materials and Methods

### Study samples

10 *ml* blood samples were obtained from 10 healthy donors in Tabriz University of Medical Sciences. Human samples were collected upon Tabriz University of Medical Sciences review board approval and patient written informed consent. Peripheral Blood Mononuclear Cells (PBMCs) were isolated by Ficoll- Hypaque (Pharmacia) gradient centrifugation of buffy coats.

### Cell Culture

Human Embryonic Kidney 293 TLR2 (HEK 293 TLR2) was cultured in complete RPMI 1640 medium (Gibco BRL, USA) supplemented with 5% heat inactivated Fetal Bovine Serum (FBS) 100 *units/ml* penicillin, 100 *units/ml* streptomycin. Peripheral venous blood mononuclear cells were collected in heparin-containing tubes. Human PBMCs were isolated by Ficoll-Hypaque (Pharmacia) gradient centrifugation of buffy coats from healthy donors and suspended in complete RPMI 1640 medium containing 10% FCS, 100 *U/ml* penicillin and 100 *mg/ml* streptomycin. All cells were maintained in a humidified incubator with 5% CO_2_ at 37°*C*. HEK 293 TLR2 and PBMCs were pre-stimulated by overnight incubation with 1 *μg/ml* LPS (Sigma-Aldrich, St. Louis, MO, USA; 0111:B4).

### MTT cell proliferation assay

HEK 293-TLR2 cells were seeded at density of 15×10^3^ cells/well in a 96-well tissue culture plate. The following day media were replaced and 200 *μl* of fresh media containing various concentrations of M2000 (2.5, 5, 25, 100, 200, 400 *μg/ml*) were added. Incubation times included 24, 48 and 72 *hr* at 37°*C* and 5% CO_2_. Then, the media were removed and the cells were washed by Phosphate Buffered Saline (PBS), and then 200 *μl* of 5 *mg/ml* MTT solution was added to every well and incubated for 4 *hr* at 37°*C* with 5% CO_2_. The solution was removed and dimethyl sulfoxide (DMSO) (sigma) was replaced instead to dissolve formazin crystals. Eventually, the plates were read at 570 *nm* in a micro titer plate reader. Drug concentrations were determined using half maximal Inhibitory Concentration (IC50) results from dose- response curves. The formula for calculating cell variability percentage is optical density of treated cells divided by the absorbance of control cells multiplied by 100.

### Cell treatment

PBMCs and HEK-TLR2 cells were cultured in 6-well cell culture plate at 5×10^5^. According to IC50 range, 5 and 25 *μg/ml* concentrations were considered for cell treatment in 24 *hr* and the cells were treated by 5 and 25 *μg/ml* of M2000 in duplicates. TLR2 signaling pathways were activated by use of 1 *μg/ml* LPS (Invivogen, USA) for 24 *hr*. Cells were located in four categories including no treated cells, cells plus different concentrations of drug, cells plus LPS and cells with drug and LPS.

### RNA extraction and quantitative real time PCR

HEK TLR2 cells were lysed in TRIzol reagent (Invitrogen) and total RNA isolation was completed according to the manufacturer’s instructions. Isolated PBMCs were used for miRNA-155 extraction by means of miRNeasy Mini kit (Qiagen) according to the manufacturer’s protocol. The RNA concentration was determined with NanoDrop Spectrophotometer (Nanodrop Technologies). cDNA was synthesized from equivalent amounts of RNA with MystiCq™ microRNA cDNA Synthesis Mix kit. Quantitative real-time PCR was performed in a Corbett Rotor-gene 6000 machine with primer sets for SOCS1, SHIP1 and β-actin (Table 1). Ratio of SOCS1 And SHIP1/β-actin was calculated with 2^−ΔΔCt^, and β-actin was used as an internal control. Real-time PCR was designed as followed: initial denaturation for 2 *min* at 95°*C*, denaturation for 20 *s* at 95°*C*, 40 cycles of annealing for 30 *s* at 58°*C* and extension for 20 *s* at 72°*C*. For analysis of miR-155, total RNA was polyadenylated, reversed transcript to cDNA by using miScript Reverse Transcription Kit (Sigma). Amplification of synthesized cDNA was carried out by real time qPCR using the SYBR Green master mix, a universal reverse primer (Ambion) and amiR-155-specific forward primer (both reagent from Qiagen, Hilden, Germany). Sample data were normalized to the U6 snRNA level (as an internal control) and are presented as fold change relative to unstimulated cells (set at one fold). Data are shown as the mean±SD of three experiments. The sequences of the primers and real time qPCR condition are listed in table 1.

**Table 1.** Primer sequences and real time qPCR conditions

**Primer**	**Sequence**
**SOCS1**	
	Reverse: TCCGGGGGTGCGCTGGC
	Forward: AGCTGCACGGCTCCTGGC
**SHIP1**	
	Reverse: GCCTTCGGATGCCTGAACA
	Forward: TCTGCGTGCTGTATCGGAA
**β-Actin**	
	Reverse: AGCACTGTGTTGGCGTACAGGTC
	Forward: AGCCTTCCTTCCTGGGCATGG
**U6**	5′-UACUUUAGUACUAGUACAAAUC-3′
**Universal primer**	5′-UGAGAACUGAAUUCCAUGGGUU-3′
**miR-155**	5′-UUAAUGCUAAUCGUGAUAGGGGU-3′

	**Denaturation**	**Annealing**	**Extension**	**Number cycles**
β**-Actin**	95 c/20 *s*	58c/30 *s*	72c/20 *s*	40
**SOCS1**	95 c/20 *s*	60c/ 30 *s*	72c/20 *s*	40
**SHIP1**	95 c/20 *s*	59c/ 30 *s*	72c/20 *s*	40
**miR-155**	95 c/20 *s*	58c/30 *s*	72c/20 *s*	40
**U6**	95 c/20 *s*	58c/30 *s*	72c/20 *s*	40

### Statistical analysis

Data are expressed as mean±SD using Graph pad prism 6 software and one-way ANOVA. P-values below 0.05 were regarded as statistically significant. Star attached (*) to indicate p<0.05 and two stars (**) to indicate p<0.01.

## Results

### M2000 MTT assay results

**Figure 1.**
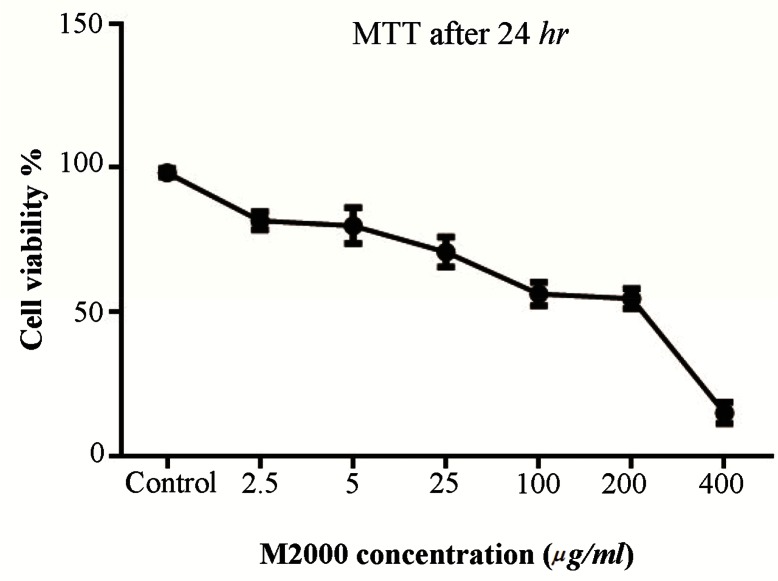
HEK-293 TLR2 MTT assay. HEK-293 TLR2 cells were treated with different concentrations of M2000 for 24, 48 and 72 *hr*. In accordance with the results, 5 *μg/ml* and 25 *μg/ml* were determined for treating cells as low and high dose.

HEK 293 TLR2 cells were treated by M2000 at 2.5, 5, 25, 100, 200 and 400 *μg/μl* doses. Cell cytotoxicity was evaluated 24, 48, 72 *hr* after treatment by drug time and dose dependently. IC50 of M2000 was 25 *μg/ml* in 24 *hr* of exposure (Figure 1).

### miR-155 expression level in M2000 treated PBMC

**Figure 2.**
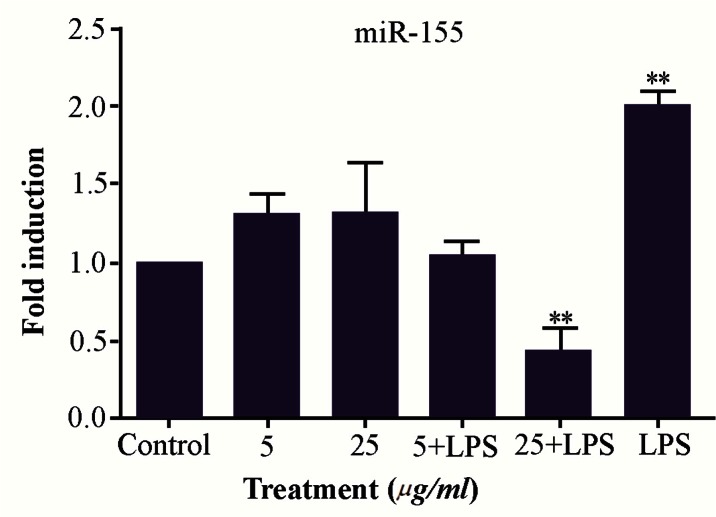
LPS and M2000 effects on miR-155 expression level in treated PBMCs. Expression level of miR-155 increased in LPS (1 *μg/ml*) treated PBMCs. M2000 significantly reduced miR-155 expression level in LPS-treated cells.

miR-155 expression profile was measured in the PBMCs with different doses of M2000 (5, 25 *μg/ml*), activated with LPS and cells were treated with LPS and M2000 using real-time qPCR. Our results showed that LPS increased expression level of miR-155 in LPS treated cells 1.99 fold (1.999±0.096, p<0.0001) in comparison with control group. Also co-treatment of LPS and high dose of M2000 compared to LPS treated cells decreased expression level of miR-155 2.39 fold (0.41±0.1516, p<0.0001). In co-treatment of LPS and low dose of M2000, miR-155 expression level decreased 0.964 fold (1.03±0.096, p=0.0002) while its expression level in low and high dose of M2000 increased 1.29 fold (1.29±0.1451, p=0.18) and 1.31 fold (1.31±0.3265, p=0.1456), respectively which did not reach the statistical significance (Figure 2).

### Up regulation of SOCS1 and SHIP1 in M2000 treated cell line

SOCS1 and SHIP1 were detected by quantitative real time PCR in high and low dose of M2000 treated HEK293 TLR2 cell line with and without LPS stimulation. The expression levels of SHIP1 and SOCS1 mRNA were up-regulated in HEK TLR2 treated by high and low concentration of M2000 after 24 *hr*, compared to untreated cells.

Expression levels of SHIP1 in M2000 treated cells (5, 25) as compared to control group increased 1.68 fold (1.68±0.038, p=0.0171) and 1.89 fold (1.89±0.306, p=0.0047), respectively. The expression level in the other group which was stimulated with LPS and drug (5, 25) increased 1.53 fold (1.53±0.1607, p=0.0494) and 1.85 (1.85±0.1123 p=0.0058), and the expression level of SHIP1 in LPS activated cells decreased 2.33 fold induction (0.4278±0.1168, p=0.0307) as compared with untreated cells.

**Figure 3.**
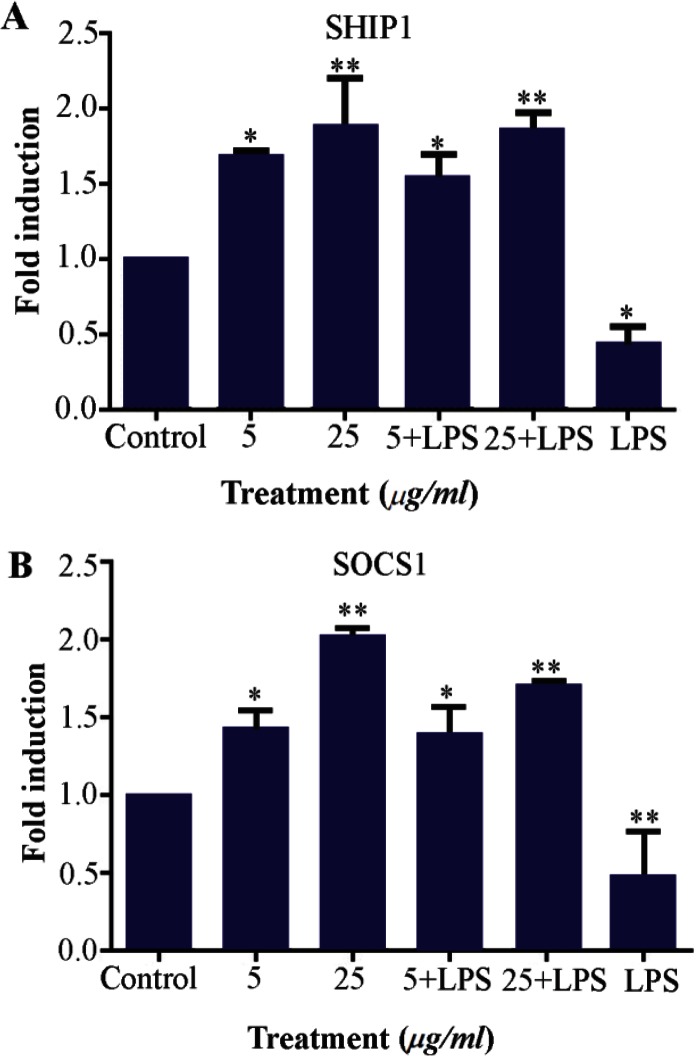
SOCS1 and SHIP1 expression levels in HEK293 TLR2 treated cells in different conditions, by LPS (1 *μg/ml*), M2000 in 5 and 25 *μg/ml* and co treated by M2000 and LPS. mRNA expression level of SHIP1 was reduced in treatment by LPS and increased in co treatment of M2000 dose dependently. (A) mRNA expression level of SOCS1 decreased in LPS treated cells and was up-regulated in different conditions of M2000 (B).

Expression level of SOCS1 at 5 *μg/ml* dose of M2000 increased 1.425(1.42±0.1207, p=0.0237) fold induction, at 25 *μg/ml* dose of M2000 increased 2.014 fold induction (2.014±0.04424, p=0.0003), co treatment of LPS with M2000 different doses (5, 25 *μg/ml*) increased 1.38 fold induction (1.389±0.1746, p= 0.0317) and 1.70 fold induction (1.70±0.0206, p= 0.0024) compared with control group. On the other hand, in LPS treated cells, expression levels decreased 2.12 fold induction (0.470±0.2635, p=0.0090) (Figure 3).

## Discussion

Investigating expression levels of SOCS1 and SHIP1, as key adaptor proteins in TLR2 signaling, it was found that M2000 could up-regulate TLR2 signaling in LPS induced inflammatory process and could affect cytokine production. TLRs are membrane-bound proteins that identify invading organisms bearing Pathogen-Associated Molecular Patterns (PAMPs) and Damage-Associated Molecular Patterns (DAMPs) 14. TLRs can be activated by PAMPs or DAMPs which lead to up regulation of inflammatory cytokines and mediators. PAMPs are conserved molecules derived from micro-organisms, such as flagellin, peptidoglycan, microbial nucleic acids and LPS 15,16. Micro RNAs (miRNAs) are small double stranded noncoding RNAs and generally have about 18–24 nucleotides which bind to the 3′ Untranslated Region (UTR) of target mRNA and direct their post-transcriptional repression 17. Overexpression of miR-155 in immune system cells can lead to down-regulation of SHIP1 and SOCS1 *via* TLR2 signaling. Consequently, Akt/NF-kB pathway is activated and production of pro-inflammatory cytokines is increased. In some inflammatory diseases, miR-155 expression was deregulated in human PBMCs 18. MiR-155 expression increases in inflammatory and auto immune conditions 19.

In inflammatory responses, up regulation of SOCS1 and SHIP1 proteins inhibits cytokine signaling *via* targeting the Janus kinase and Signal Transducer and Activator of Transcription (JAK/STAT) pathway. It has been suggested that SOCS1-deficient mice are highly sensitive to LPS-induced endotoxin shock and exhibit increased levels of inflammatory cytokines 20. Strassheim *et al* showed that SHIP-deficient (SHIP−/−) mice were hypersensitive to LPS-induced morbidity, while others demonstrated that SHIP down regulated TLR2-dependent induction of proinflammatory cytokines and chemokines 21,22. Therefore, targeting SOCS1 and SHIP1 could reduce undesirable complications in inflammatory diseases. Additionally, SOCS1 deficient

Dendritic Cells (DCs) express highly B cell Activating Factor (BAFF) which results in abnormal B cell proliferation and antibody production. Moreover, decreased suppressive capacity of Treg is related to low levels of SOCS1. These Treg cells maintain hyper activated B cells by promoting interaction of B cells with self-reactive TCD4^+^ that leads to production of inflammatory cytokine and diverse autoantibodies. On the other hand, up regulated levels of SOCS1 created by JAK/ STAT signaling maybe serve as protective molecules in controlling destructive inflammatory cytokines. Also, down regulation of SHIP-1 leads to a decrease in levels of phosphorylation of Akt and probably as a consequence, the NF-kB pathway activation. Here, it was found that M2000 significantly up regulates the relative expression of SOCS1 and SHIP1 and thus can improve adverse complications of inflammatory and auto-immune diseases.

O’Connell *et al* revealed that miR-155 was up-regulated consistently in the mammalian inflammatory response. Ceppi *et al*’s results demonstrated that miR-155 exerts negative effects on inflammation by alternative negative feedback pathway *via* acting on the TLR/IL1-R inflammatory pathway. MiR-155 inhibited activation of transforming growths factor beta-activated kinase1 (TAK1) by targeting TAK1 binding protein2 (TAB2), and hence NFκB and MAPK; thus acting as an anti-inflammatory agent. On the other hand, Tili *et al* has shown that miR-155 expression likely has positive effects on the NF-κB signaling proteins by acting post transcriptionally to regulate the levels of different proteins involved in this pathway, including TNF-α. So that miR-155 positively regulates the production of inflammatory cytokines during the innate immune response. In this regard, it was shown that LPS could induce expression level of miR-155 in treated cells and M2000 is able to reduce expression level of miR-155 in these cells. So, M2000 has key role in management of inflammation process by targeting miR-155.

Mirshafiey *et al* showed that biocompatibility and tolerability property of M2000 is higher than conventional NSAIDs and in spite of the pharmacological effects of M2000, there have been no systematic toxicological studies on its safety so far. Anti-inflammatory and immunosuppressive properties of M2000 were shown in our previous study 23. For example, in nephritis M2000 reduced inflammatory reactions *via* diminished antibody production and urinary protein excretion in treated rats versus non-treated control. Also, in the experimental model of multiple sclerosis, using M2000, matrix metalloproteinase activity was suppressed, which is parallel with immunoprotective mechanism of IFN-beta 4,6,24.

Taking together, M2000 can be defined as a novel NSAID and a new candidate in treatment of inflammatory and autoimmune diseases. It is recommended to conduct *in vivo* studies by using animal models and utilize other cell lines for evaluation of expression level of miR155 and these targets. Moreover, there are several PRRs that must be assessed for further characterization of M2000 signaling pathway.

## Conclusion

Our results showed that LPS reduced expression levels of SOCS1 and SHIP1 in LPS treated cells, which result in LPS-induced inflammation. Moreover, M2000 ability in SOCS1 and SHIP1 up-regulation was shown. Additionally, our finding revealed that M2000 was able to decrease expression level of LPS-induced miR-155 in LPS and M2000 co-treated cells condition. So, M2000 can be used as a new candidate in treatment of inflammatory diseases by targeting downstream adaptor molecules of TLR2 signaling pathway along with miR-155.
